# Overcoming extended lag phase on optically pure lactic acid production from pretreated softwood solids

**DOI:** 10.3389/fbioe.2023.1248441

**Published:** 2023-09-07

**Authors:** Joana Campos, Henrik Almqvist, Jie Bao, Ola Wallberg, Gunnar Lidén

**Affiliations:** ^1^ Department of Chemical Engineering, Lund University, Lund, Sweden; ^2^ CR Competence AB, Lund, Sweden; ^3^ State Key Laboratory of Bioreactor Engineering, East China University of Science and Technology, Shanghai, China

**Keywords:** D-lactic acid, *Pediococcus acidilactici*, softwood, cell-solid interaction, lag phase, inhibition

## Abstract

Optically pure lactic acid (LA) is needed in PLA (poly-lactic acid) production to build a crystalline structure with a higher melting point of the biopolymer than that of the racemic mixture. Lignocellulosic biomass can be used as raw material for LA production, in a non-food biorefinery concept. In the present study, genetically engineered *P. acidilactici* ZP26 was cultivated in a simultaneous saccharification and fermentation (SSF) process using steam pretreated softwood solids as a carbon source to produce optically pure D-LA. Given the low concentrations of identifiable inhibitory compounds from sugar and lignin degradation, the fermentation rate was expected to follow the rate of enzymatic hydrolysis. However, added pretreated solids (7% on weight (w/w) of water-insoluble solids [WIS]) significantly and immediately affected the process performance, which resulted in a long lag phase (more than 40 h) before the onset of the exponential phase of the fermentation. This unexpected delay was also observed without the addition of enzymes in the SSF and in a model fermentation with glucose and pretreated solids without added enzymes. Experiments showed that it was possible to overcome the extended lag phase in the presence of pretreated softwood solids by allowing the microorganism to initiate its exponential phase in synthetic medium, and subsequently adding the softwood solids and enzymatic blend to proceed to an SSF with D-LA production.

## 1 Introduction

The starch-based production of lactic acid (LA) from bio-based raw materials has been important in the success of poly-lactic acid (PLA) as a replacement for fossil-based plastics. LA is one of the most successful examples of industrial large-scale microbial fermentation, and it is furthermore the best way to obtain optically pure LA, without relying on downstream processing to separate both enantiomers—L-LA and D-LA (Yi et al., 2016; de Oliveira et al., 2022; Lomwongsopon and Varrone, 2022; Ojo and de Smidt, 2023). The access to pure LA enantiomers makes it possible to tailor ratios of poly-L-LA and poly-D-LA in a PLA polymer, giving advantages such as decreasing brittleness and the slow crystallization of PLA (Abdel-Rahman and Sonomoto, 2016; Rosenboom et al., 2022). There are bioplastic alternatives to most fossil-based plastics, such as polyhydroxyalkanoates (PHA) and bio-polybutylene succinate (bioPBS), but all bio-based and biodegradable options are still more expensive (ranging from 1.5 to 4 times) than fossil-based polymers (Lomwongsopon and Varrone, 2022; Rosenboom et al., 2022; Costa et al., 2023; Yu et al., 2023).

The use of non-food feedstocks (e.g., food, industrial or agricultural waste) instead of starch-based feedstocks (e.g., corn, sugar cane, cassava) may become a strategy to make biopolymer production processes more economically competitive (Abdel-Rahman and Sonomoto, 2016; Abedi and Hashemi, 2020; Abedi and Hashemi, 2020; de Oliveira et al., 2022). Approximately two-thirds of the Swedish land area is forest, with close to 20 million hectares used for production of wood-based products, of which 80% are softwood species (Ekström and Hannerz, 2021). The forest industry is one of the most important sectors in the Scandinavian economy, and therefore it is important to find added-value applications to its wastes. These wood residues can be upcycled as substrates for bio-based industries, to be used to produce diverse platform chemicals, such as LA.

Probably, the biggest challenge in using softwood as raw material for bioprocesses is its high recalcitrance, in comparison to other agricultural wastes (Zhu and Pan, 2010; Galbe and Wallberg, 2019). It is necessary to pretreat wood with chemical and physical methods to disrupt the structure of hemicellulose, cellulose, and lignin, making it more accessible for enzymatic hydrolysis to monomeric sugars (Galbe and Wallberg, 2019; Yankov, 2022). This pretreatment will lead to the formation of degradation products that can be inhibitory for microorganisms in the bioprocess (Liu et al., 2013; van der Pol et al., 2016; Alves de Oliveira et al., 2018). *Pediococcus acidilactici* ZP26 is a D-LA and exopolysaccharides (EPS) producer, homofermentative, microaerophilic, with proven resistance to lignocellulosic degradation products (Liu et al., 2013; Yi et al., 2016; Campos et al., 2021; 2023). This strain has been reported to produce almost 77 g/L D-LA from pretreated and biodetoxified corn stover (Yi et al., 2016). In that study, Yi and collaborators ran a 25% (w/w) solid content simultaneous saccharification and fermentation (SSF, without separation of the liquid and solid fraction of the pretreated corn stover) with 6 h of pre-hydrolysis, achieving a yield of approximately 60% on cellulose content. Upon inoculation, the biomass started producing LA directly, without any lag phase for adaptation. This was enabled by a combination of three factors: first, the decrease of the concentration of inhibitors during a biodetoxification step of the corn stover hydrolysate with *Amorphotheca resinae* ZN1; second, the inherent resistance of *P. acidilactici* ZP26 to any leftover of these lignocellulose degradation products; and finally by the high sugar availability resulting from the pre-hydrolysis of cellulose (Yi et al., 2016).

In the present study, a SO_2_-catalysed steam-explosion pretreatment was applied to spruce chips. The application of this physicochemical pretreatment in softwood has been extensively investigated since it favors fiber separation and the hydrolysis of hemicelluloses. The gaseous SO_2_ is preferred to a liquid acid since it is more evenly distributed through the biomass (Alvira et al., 2010; Galbe and Wallberg, 2019). The high temperature and low pH lead to the formation of sugar and lignin degradation products. The influence of inhibitors present in the filter-pressed liquid fraction of pretreated softwood in the production of optically pure LA was presented in previous work (Campos et al., 2023). Enzyme and cell culture inhibition are usually associated not only with toxic compounds produced in the lignocellulosic biomass pretreatment but also with high solids loadings (>15% solids (w/w) in the reaction medium) (Kadić et al., 2014; da Silva et al., 2020). However, the first SSF tests showed an unexpectedly long lag phase between inoculation and exponential LA production, even though the usual inhibitors (organic acids and furans) were not detected, and solid loadings were kept low (7% on weight [w/w] of water-insoluble solids [WIS]). The focus in the current work was therefore to find methods to decrease the lag phase experienced after the addition of pretreated softwood solids at the beginning of the fermentation time (0 h) and propose a hypothesis for this extended lag phase.

## 2 Methods

### 2.1 Softwood pretreatment

Spruce (*Picea abies*) was partially debarked, chipped, and stored at 4°C until further use. The dry matter content of the pieces was measured to be 50% (w/w) (Sluiter et al., 2008a). The softwood was impregnated with 2.5% (w/w) gaseous SO_2_, based on the moisture content of the raw material, in sealed plastic containers for 20 min at room temperature. It was subsequently vented for 30 min before the following physicochemical pretreatment. Steam pretreatment was performed at 210°C for 5 min in a 10 L reactor (Process- & Industriteknik AB, Kristianstad, Sweden) (Palmqvist et al., 1996; Frankó et al., 2019). The pretreated solids were separated from the liquid fraction on a filter press (HP25M, Fischer Maschinenfabrik GmbH), and stored at 4°C until further use. The pretreated solids had a total solids content of 41.6% (Sluiter et al., 2008a) and 38.4% (w/w) WIS (Sluiter et al., 2008b). The pretreated solids were not washed before being used in the experiments. Fermentable sugars added to the reactors were of two kinds: the glucan fraction (88.2% [w/w] WIS) and the water-soluble glucose and mannose in the interstitial liquid fraction. For the amount of non-washed pretreated solids added to the reactors (7% [w/w] WIS), approximately 4 g/L of mannose and 3 g/L of glucose came from the interstitial liquid fraction.

### 2.2 Microorganism

D-LA producer *Pediococcus acidilactici* ZP26 (Chinese General Microorganisms Collection Center (CGMCC), registration number 8665) was engineered from wild-type *P. acidilactici* DQ2, an isolate from corn stover slurry (Zhao et al., 2013), by knockout of the *ldh* gene (Yi et al., 2016).

*P. acidilactici* ZP26 was grown on simplified Man-Rogosa-Sharp (MRS) medium: 10.0 g yeast extract, 10.0 g peptone, 5.0 g sodium acetate, 2.0 g ammonium citrate dibasic, 2.0 g dipotassium phosphate, 0.58 g magnesium sulfate heptahydrate, 0.25 g manganese sulfate monohydrate in 1 L of demineralized water (Yi et al., 2016). Glucose at 500 g/L was prepared in demineralized water, autoclaved, and supplemented to the simplified MRS medium in sterile conditions up to the desired concentration. All the solutions were autoclaved at 121°C for 20 min. The organism was stored at -80°C in 15% (v/v) glycerol, in 1.5 ml aliquots, which were used for inoculum preparation.

### 2.3 Cultivations before the evolutionary adaptation of *P. acidilactici*

Two 2 L Biostat A plus bioreactors (Sartorius AG, Germany) were used for non-aerated cultivations at 42°C, stirring rate of 150 rpm. Simplified MRS medium and carbon source were added up to 1 kg, according to the chosen conditions for each test. The reactors were run for 96 h or until the carbon source was depleted. The conditions are described in depth in the results and discussion sections. pH was maintained at 5.5 by automatic titration using a 2 M NaOH solution. All reactors with pretreated solids were run at 7% (w/w) WIS added at inoculation. In some cases, glucose was supplemented to the reactors, in the appropriate concentration. The enzymatic hydrolysis of the solids was performed with Cellic CTec3, a commercial cellulase and hemicellulase mixture, kindly provided by Novozymes (Denmark). To assess the influence of enzyme concentration, 3.5 g or 17.5 g of Cellic CTec3 blend were added to the broth, corresponding to 7.5% and 38% g Cellic CTec3/g cellulose, respectively. In the reactors with pre-hydrolysis, conditions were identical to those used during cultivation: 42°C, pH 5.5, and 150 rpm. Materials and the simplified MRS medium were sterilized at 121°C for 20 min. The seed culture was started with 0.5 ml of stock culture in 120 ml simplified MRS medium, supplemented with 20 g/L glucose and 0.6 g of CaCO_3_ per g of glucose as a pH buffer. The inoculum was grown for 12 h at 150 rpm and 42°C, in an orbital shaker New Brunswick Innova 40. Each bioreactor was inoculated with 10% (v/v) of the seed culture. Samples were regularly withdrawn for pH measurement and chromatographic analyses. All conditions were tested in duplicate.

### 2.4 Evolutionary adaptation of the strain

During the previously described tests on SSF with and without pre-hydrolysis, it was verified that the microorganism presented lower LA-specific production rates compared to previous studies (Campos et al., 2021; Campos et al., 2023), probably related to loss of activity of the organisms stored at -80°C in 15% (v/v) glycerol for 4 years. *P. acidilactici* ZP26 was therefore evolutionarily adapted by the continuous transfer of culture broth onto fresh medium. Briefly, 1% (v/v) of the culture broth from the previous cultivation was transferred to fresh simplified MRS medium, with glucose and mannose in equal concentration as carbon source (10 g/L), to promote simultaneous consumption of both sugars, described in previous work (Campos et al., 2021). The transfers occurred every 24 h and incubated at 42°C and 150 rpm, and a total of 9 transfers were performed. The final culture was then stored at -80°C in 15% (v/v) glycerol, in 1.5 ml aliquots, which were used as inoculum for the following tests.

### 2.5 Cultivations after evolutionary adaptation of *P. acidilactici*

Cultivations were conducted in 2 L Biostat A plus bioreactors (Sartorius AG, Germany), at 42°C, stirring rate of 150 rpm, no aeration, and pH was maintained at 5.5 by automatic titration with 2 M NaOH. As in the previous tests, simplified MRS medium and carbon source were added up to 1 kg, according to the chosen conditions for each test. The reactors were run for 96 h or until the carbon source was depleted. The inoculum was grown as described before, using the newly adapted stock cultures as a starter, and 10% (v/v) was used as seed culture for each test. Each run is described in detail in the results section of this study. Cultivations were run with either no addition of solids, the addition of 7% (w/w) WIS in pretreated softwood solids, a mixture of 30 g/L Kraft lignin (CAS 8068-05-1 from Sigma-Aldrich) and 45 g/L Avicel^®^ PH-101 cellulose (CAS 9004-34-6, from Sigma-Aldrich), or addition of 36.8 g of dried spruce sawdust, which is equivalent to 7% (w/w) WIS. Supplementation with 40 g/L glucose was tested for some of the conditions. In the case of the test with enzymatic hydrolysis of the pretreated solids (Glucose & SSF at 3 h), only 20 g/L of glucose were added since the initial concentration of free glucose might influence the performance of the enzymatic cocktail. To exclude the influence of enzyme concentration, 38% (g enzyme blend/g cellulose) of Cellic CTec3 was added to the broth, corresponding to 17.5 g. Tween 80 (1% v/v) was added to a SSF test, to evaluate the effect of a surfactant in the production of D-LA from pretreated softwood solids. Materials and simplified MRS medium were sterilized at 121°C for 20 min. Samples were regularly withdrawn for pH measurement and chromatographic analyses. All conditions were tested in duplicate.

### 2.6 Analyses

Softwood pretreated solids were characterized according to the Laboratory Analytical Procedures of the National Renewable Energy Laboratory for the determination of total solids (Sluiter et al., 2008a), determination of insoluble solids (Sluiter et al., 2008b) and determination of structural carbohydrates and lignin in biomass (Sluiter et al., 2012).

Samples were centrifuged at 12,000 × *g* for 3 min, filtered with coupled 0.45 µm and 0.2 µm syringe filters because of the solid particle amount in suspension and stored at −20°C until HPLC analysis. The HPLC analysis was conducted in an Agilent Technologies HPLC, model 1,260 Infinity II, comprised of a module with an isocratic solvent pump, autosampler, column oven, UV detector, and RI detector. Monosaccharides (glucose, xylose, mannose, arabinose), organic acids (succinic acid, lactic acid, formic acid, acetic acid, levulinic acid), hydroxymethyl-furfural (HMF) and furfural were analyzed with a Hi-Plex H (Agilent Technologies, CA, United States ) column at 65°C, using 5 mM sulfuric acid as eluent at 0.6 ml/min. Lactic acid isomers were separated on a Supelco Astec CLC-D (Merck, Darmstadt, Germany) column run at room temperature, with 5 mM copper sulfate in demineralized water as eluent at 1.0 ml/min. The concentration of the sugars in the enzymatic cocktail was determined by HPLC analysis and accounted for in the presentation of the results.

## 3 Results

### 3.1 Identification of a lag phase in D-LA production when using pretreated softwood solids

Inhibition of fermentation is most often caused by inhibitors present in the liquid fraction (Zhang et al., 2010; Yi et al., 2016; Qiu et al., 2020). In a previous study, we have shown promising results on D-LA production from the liquid fraction of the pretreated softwood (Campos et al., 2023). In the current work, the focus was shifted to the utilization of the pretreated solids from the softwood. The hexose sugars, predominantly glucose but also some remaining mannose, are released through enzymatic hydrolysis of the solids after pretreatment (consisting of cellulose and remaining hemicellulose not hydrolyzed in the pretreatment as well as lignin), which is retained together with lignin in the solids after pretreatment.

**FIGURE 1:**
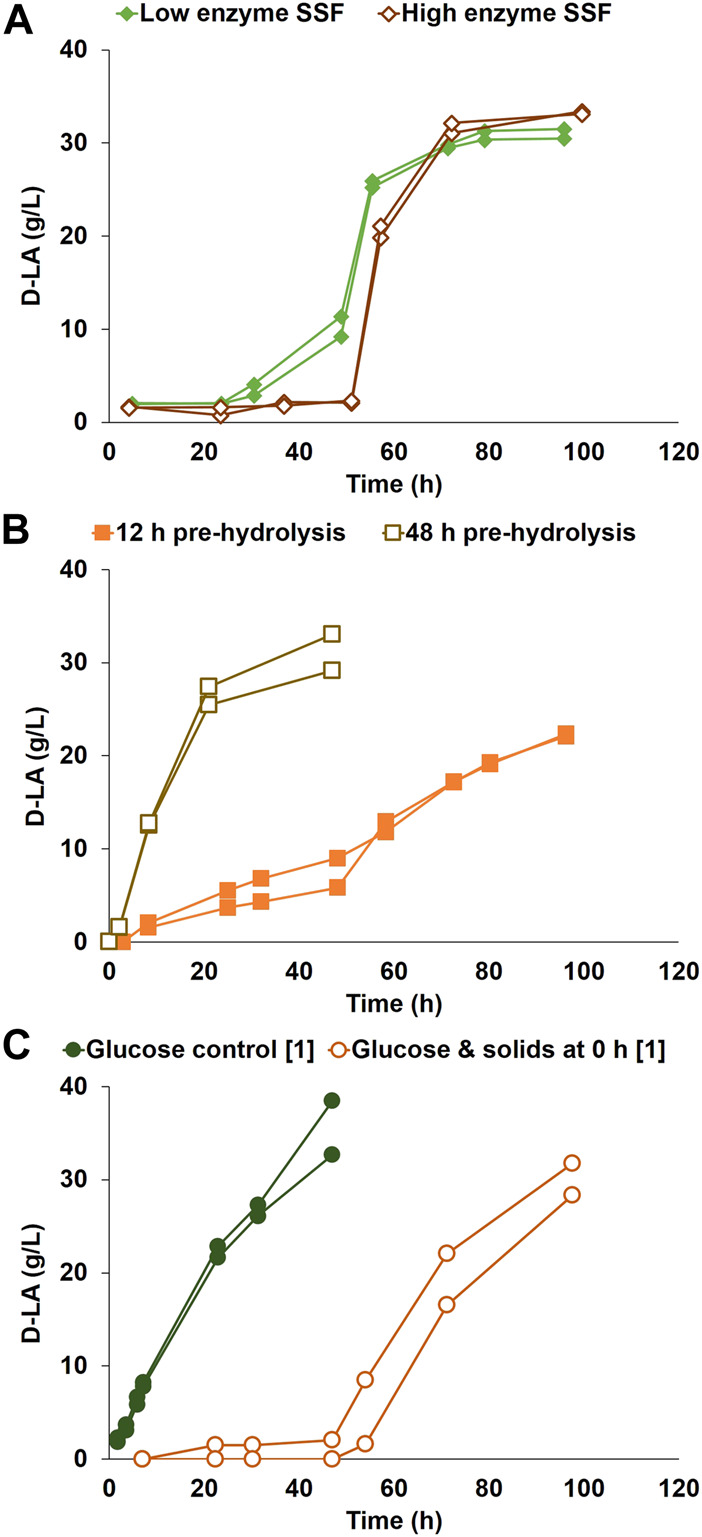
D-LA production by *P. acidilactici* ZP26 on **(A)** SSF with low [3.5 g] and high [17.5 g] enzyme content, using pretreated softwood solids as sole carbon source, without pre-hydrolysis **(B)** SSF after 12 h and after 48 h of pre-hydrolysis, using pretreated softwood solids as sole carbon source and 3.5 g of enzymatic blend **(C)** glucose control, and bioreactor duplicate with glucose solution and pretreated softwood solids added at inoculation, without enzymatic blend. The conditions marked with [1] were repeated after strain evolutionary adaptation. Duplicates are represented as two lines in the same color and marker.

SSF was run with low and high enzyme content, 3.5 g and 17.5 g Cellic CTec3 (Figure 1A). For the cultivations with low enzyme content, D-LA production started at a low rate after a delay of 24 h, with an increase in the volumetric production rate to a maximum of 2.32 g/(L·h) at around 45 h, reaching a maximum D-LA concentration of 31 g/L. Higher enzyme loadings led to a slightly higher final D-LA titer (33 g/L), but the production did not start until after 50 h, even though plenty of sugars were available in less than 12 h (approximately 40 g/L of glucose). The maximum volumetric D-LA production rate was 2.96 g/(L·h) during the exponential phase.

Yi et al. (2016) previously reported SSF conversion of corn stover using *P. acidilactici* ZP26, achieving 76.8 g/L D-LA at high optical purity. The delay in D-LA production in the SSF of the detoxified corn stover was much shorter than the one found for pretreated softwood solids in the current work. However, in the work by Yi et al., a pre-hydrolysis of 6 h was used. A set of experiments with pre-hydrolysis, of either 12 or 48 h, was therefore made to see the effect of initial glucose concentration in the medium and possible changes in the structure of the solids during the lag phase (Figure 1B). The cultivations with 12 h pre-hydrolysis had a lower D-LA volumetric production rate until 48 h after inoculation (0.14 g/(L·h)), from which time the rate doubled. In contrast, D-LA production started right after inoculation for the tests with 48 h of pre-hydrolysis at a volumetric production rate of 1.26 g/(L·h). The D-LA titers were lower for 12 h pre-hydrolysis (an average of 22 g/L) in comparison to 48 h pre-hydrolysis (31 g/L).

Further experiments were made to determine the effect of initial glucose *per se* on the lag phase, in which glucose was added at the beginning of the fermentation (Figure 1C). No enzymes were added, which meant that cellulose fibers were not hydrolyzed. A glucose control was also run. Similarly to the SSF experiments, D-LA production did not start until after a long lag phase of approximately 48 h when pretreated softwood solids were present. After that lag phase, the D-LA volumetric production rate reached a maximum of 0.75 g/(L·h), with the control reaching 0.96 g/(L·h), with average D-LA final concentrations of 30.0 g/L and 35.6 g/L, respectively.

### 3.2 Overcoming the inhibition by pretreated softwood solids

A lag phase of up to 48 h is highly undesirable and must be avoided in an industrial SSF process. The lag phase may be shortened, or avoided, by use of a higher inoculum density, by adding medium components, or by using cells in a different physiological state, and these strategies were investigated here.

**FIGURE 2:**
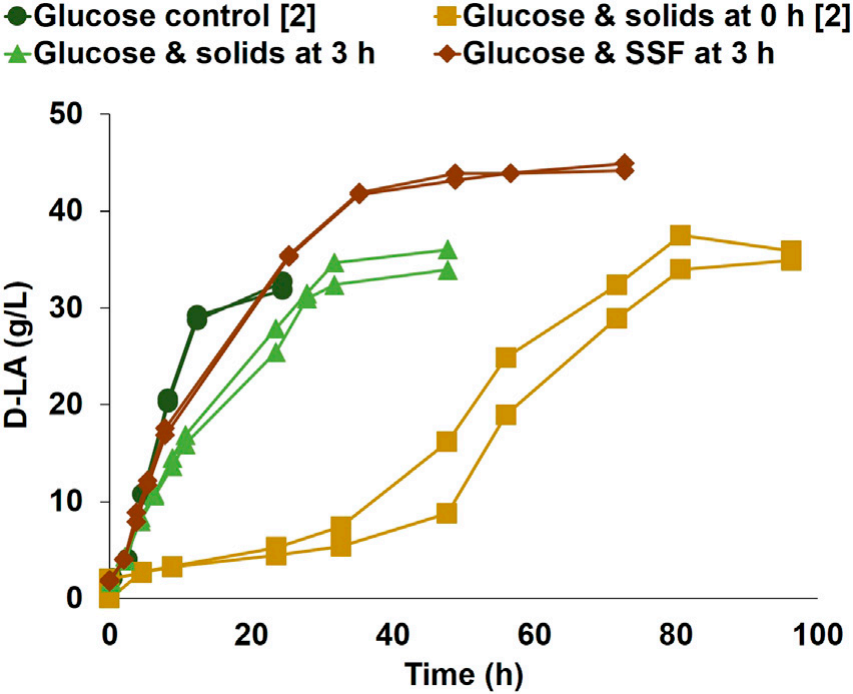
D-LA production by *P. acidilactici* ZP26 on a glucose control; bioreactor duplicates with glucose solution and pretreated softwood solids added at inoculation (0 h), without enzyme blend; bioreactor duplicate initiated on glucose solution, with the addition of pretreated softwood solids at 3 h of fermentation, without enzyme blend; bioreactor duplicate initiated on glucose solution, with the addition of pretreated softwood solids and enzymes at 3 h of fermentation, on SSF mode. The conditions marked with [2] were performed after strain evolutionary adaptation. Duplicates are represented as two lines in the same color and marker.

When comparing the results in Figure 1C with previous work (Campos et al., 2021), it was found that the glucose consumption and D-LA production volumetric rates in the glucose control were lower than expected (0.94 g/(L·h), in comparison with previously reported 1.39 g/(L·h)). An evolutionary adaptation of the frozen stock cultures of *P. acidilactici* ZP26 was therefore conducted before further studies on the reason for the extended lag phase. After the evolutionary adaptation, the D-LA volumetric consumption for glucose controls with 1% and 10% (v/v) of inoculum were 2.77 g/(L·h), and 2.54 g/(L·h), respectively. The larger inoculum showed, however, no lag phase, while a lower inoculum needed approximately 2 h to start its exponential phase (Figure 2). The previously tested cultivation with glucose and non-hydrolyzed solids (Figure 1C) was repeated with the adapted strain and higher inoculum density. The results (Figure 2, Glucose and solids at 0 h) were very similar to those shown in Figure 1C, with a long lag phase of 40 h and D-LA volumetric production rate of 0.70 g/(L·h), i.e. the evolutionary adaptation did not solve the problem of the extended lag phase.

One important question was if the long lag phase was due to solids *per se* or due to the specific nature of the solids, e.g. presence of interstitial inhibitors or adsorption properties of the particles. For this reason, more trials were conducted using a synthetic glucose solution as a carbon source, and supplementing the medium with sawdust, or a mixture of model cellulose (Avicel) and lignin (Kraft lignin) (see Supplementary Figure S2). None of these cultivations showed any appreciable lag phase and D-LA production started right after inoculation. Notwithstanding, production rates were lower than the glucose control (Figure 2), at 1.67 g/(L·h) and 1.92 g/(L·h), respectively.

The influence of the cell mass development at the start of D-LA production was examined by adding pretreated solids to exponentially growing cells (Figure 2, Glucose and solids at 3 h). Here, inoculation was made into the reactor, which contained 40 g/L of glucose as a carbon source but no solids or enzyme. The growth started right after inoculation, and the addition of only pretreated softwood solids at 3 h did not interrupt the exponential phase of D-LA production. The maximum volumetric production rate was 1.06 g/(L·h) and the final D-LA titer averaged 35.0 g/L.

It was important to verify if these positive results would be maintained while using the pretreated softwood solids as a carbon source for D-LA production, and therefore the addition of both pretreated softwood solids and enzyme blend (17.5 g Cellic CTec3 blend) was made at 3 h of cultivation (Figure 2, Glucose & SSF at 3 h). The glucose concentration was lower in this experiment (20 g/L) to limit the inhibitory effect of a high glucose concentration on the performance of the enzymes (da Silva et al., 2020). A similar profile to the cultivations with the addition of pretreated solids without enzyme was observed. After accounting for the glucose initially present, the sugars from the enzymatic cocktail, and glucose liberated by enzymatic hydrolysis, it was confirmed that softwood was used for D-LA production, reaching a final D-LA titer of 44.5 g/L at a maximum volumetric rate of 2.26 g/(L·h). These experiments reinforced that it was possible to, statistically (two-sample *t*-test), decrease significantly (t (18) = 24.8, *p* = 1.2 × 10^−15^) the lag phase time between the cultivations with solids added at 0 h of fermentation (mean = 48, standard deviation = 4.5) and the cultivations with solids added at 3 h of fermentation (mean = 0, standard deviation = 0).

SSF reactors using pretreated softwood solids as a carbon source were also conducted using 1% (v/v) Tween 80 (see Supplementary Figure S2), in an attempt to reduce the lag phase by reducing interaction between cells and fibers. Tween 80 is part of the original recipe for the MRS medium, and has been reported to positively affect enzymatic hydrolysis by blocking the adsorption of enzymes to non-productive sites (Jørgensen et al., 2007; Hou et al., 2022; Huang et al., 2022). The use of the surfactant decreased the time to the start of the exponential phase to approximately 24 h, with a volumetric production rate of 1.10 g/(L·h). The effect of Tween 80 on the lag phase was not related to the enzymatic hydrolysis but likely related to effects on the interaction between the surface of the pretreated softwood solids and the bacterium.

## 4 Discussion

An unexpected delay in the start of D-LA production from pretreated softwood solids by *P. acidilactici* ZP26 was found in the present work (Figure 1A). The presence of pretreated softwood solids led to a longer delay, whether or not the solids were used as carbon source. In this study, a lag phase was defined as the time from inoculation until the beginning of the exponential phase. In the search for the reason for such a long lag phase, the addition of Tween 80 to the pretreated softwood solids was found to decrease the lag phase by half (see Supplementary Figure S2). No, or only a short, lag phase (0–4 h after inoculation) was observed in glucose controls. The addition of other solid materials, such as a mixture of Avicel cellulose and Kraft lignin, or sawdust, also gave no delay in the start of the exponential production of D-LA (see Supplementary Figure S2). Very importantly, there was no lag phase if pretreated solids were added to exponentially growing cells (3 h after inoculation on glucose). Extensive (pre-)hydrolysis of the pretreated softwood solids (48 h) also removed the lag phase, but a substantial analogous phase of 40 h or more was observed with a pre-hydrolysis time of 12 h. This suggests that there was an interaction between pretreated but not enzymatically hydrolyzed fibers and the stationary cells causing an extended lag phase.

The addition of a higher enzyme concentration to the SSF led to faster substrate availability but did not decrease the 40-hour-long lag phase (Figure 1A). This was followed by tests with pre-hydrolysis of the solids (Figure 1B), which would make cellulosic glucose readily available for consumption after inoculation. 12 h of pre-hydrolysis led to a slow production period soon after inoculation, while the volumetric production rate was still very low in comparison with other conditions. This low production period was not caused by a lack of enzyme activity, as shown by measurements of glucose concentrations in the broth, as assessed by measurement of the glucose concentration in the broth (see Supplementary Figure S1). In these reactors, the glucose concentration in the medium started to decrease rapidly at 48 h after inoculation, while the D-LA production rate increased, which hinted at an unidentified change in the medium. To allow for maximum solids hydrolysis before inoculation, a separate hydrolysis and fermentation (SHF) mode was simulated in bioreactors with 48 h pre-hydrolysis (Figure 1B). To confirm that the longer lag phase was caused by any problem with the inoculum, control bioreactors using a glucose solution as the sole carbon source, without solids, were performed. The 48 h pre-hydrolysis tests (Figure 1B), together with the glucose control (Figure 1C), made it possible to rule out the possibility that the longer lag phase was caused by a deficient inoculum since the production of D-LA started right after inoculation for both cases.

Optimizing the hydrolysis yield of the pretreated softwood was not a target for the study. Moreover, the presence of inhibitors such as aldehydes, furans, and organic acids in aqueous solution has been demonstrated to decrease the efficiency of enzymatic hydrolysis (Palmqvist and Hahn-Hägerdal, 2000; Arora et al., 2013; Galbe and Wallberg, 2019). The tests with pre-hydrolysis left the question if the lower amount of glucose was the cause of a longer lag phase. Therefore, cultivations were run with synthetic sugars and solids, without enzymatic hydrolysis (Figure 1C). The results confirmed that the source of the monomeric glucose (synthetic medium or softwood solids) was not the reason for the longer lag phase, since bacteria were not able to quickly start consuming the monomeric sugar available.

In other studies that use softwood as a carbon source for bacteria, degradation products formed during the harsh physicochemical pretreatment are usually considered the most significant reasons source of microbial inhibition (Asada et al., 2012; Dietrich et al., 2019; Campos et al., 2023). These are usually found in the liquid fraction of the pretreated biomass, together with hemicellulose sugars. The longer lag phase observed in the first tests (Figure 1) exceeded the 2–4 h registered in previous studies with the liquid fraction of the pretreated softwood (Campos et al., 2023), despite the considerable amounts of lignocellulose degradation products present in the previous study. Some of the water-soluble inhibitors were also analyzed in the present study (succinic acid, lactic acid, formic acid, acetic acid, levulinic acid, HMF, and furfural) but the amounts were lower than the detection limits. The longer lag phase is therefore unlikely to be caused by inhibition by any of these compounds. Since most of the SSF studies are focused on the use of the whole pretreated lignocellulosic biomass, possible effects of the presence of solids in microorganisms’ metabolism are possibly masked.

From the results, it was possible to hypothesize that inoculum cells interact with non-hydrolyzed solids detrimentally in the initial stage of D-LA production. Enzymatic hydrolysis before inoculation (e.g., 48 h pre-hydrolysis) decreased the lag phase before the start of D-LA formation. Interestingly, there was a change in the interaction between the bacterial cells and the softwood solids after 40-plus hours of fermentation even without enzymatic hydrolysis (Figure 1C), which allows *P. acidilactici* to start fermenting the glucose into D-LA.

Previous studies have reported a decrease in particle size during agitation of pretreated softwood (Kadić et al., 2014). The referenced study showed that higher agitation rates (up to 600 rpm) were the cause of particle size reduction in high WIS loading (13%) spruce hydrolysis. Lower solid loadings (7% (w/w) WIS) did not show such prominent effects during the conversion from glucan to glucose and were therefore chosen for the present study to limit the high solid effect influence on bacterial performance. However, after the observation of a longer lag phase, parallels can be drawn between the challenges of working with such recalcitrant and fibrous materials. Kadić et al. (2014) observed that softwood fibrous structure is damaged by agitation in an aqueous solution increasing its surface area and the exposure of the cellulosic fibers. This aperture of the softwood structure, even without enzymatic hydrolysis, might be one of the reasons for the delayed start of fermentation of glucose into D-LA shown in Figure 1C. However, the results presented by Kadić et al. (2014) refer to higher solid loadings than the present study, and therefore conclusions cannot be directly applied to the present study. Other studies have been conducted on the rheological characterization of pretreated softwood (Wiman et al., 2011), but these were also coupled with enzymatic hydrolysis for cellulosic sugar utilization. A more thorough study on the structural placement of the pretreated softwood fibers without enzymatic hydrolysis would have to be conducted to understand its influence on cell behavior.

The potential of cell adsorption to wood solid particles has previously been shown to be a cause for microbial inhibition. Adherence of cells to surfaces can happen due to van der Waals, electrostatic, and acid-base interactions, and it is an important field of research since it is the first step for biofilm formation (Soumya et al., 2013; Munir et al., 2019). In the case of non-pretreated wood, extractives have been proven to have anti-microbial properties (Tomičić et al., 2020). Reactor experiments were conducted with sawdust (untreated spruce fine particles), using a synthetic glucose solution as a carbon source, and no inhibition was observed. *P. acidilactici* ZP26 was able to start consuming glucose for D-LA production right after inoculation (see Supplementary Figure S2). Therefore, neither wood extractives nor untreated softwood solids showed a negative influence on the culture. This indicated that the inhibition was caused by one or more characteristics of the pretreated solids. Cultivations with Avicel cellulose and Kraft lignin powder were conducted to assess the influence of model solid compounds on the process. However, these showed very similar results to tests with sawdust. It is known that lignin has a complex structure that cannot be modeled after these types of reagents. The results indicate that inhibition was not caused by chemical interactions between the cells and the lignin and cellulose molecules, or that the model Kraft lignin used is not representative of that present in the dilute-acid pretreated softwood solids.

The complex 3D structure of the pretreated softwood solids seemed to play an important role in the observed microbial inhibition. Steam explosion pretreatment opens the wood fiber structure, making cellulose more accessible to enzymes and microorganisms (Galbe and Wallberg, 2019), but also exposing lignin. Cellulolytic enzymes bind to lignin, making them useless for the enzymatic hydrolysis of lignocellulose (Pan et al., 2005). The addition of surfactants to the enzymatic hydrolysis process has been shown as a possible solution to decrease the concentration of enzyme added for increased hydrolysis yields (Kandhola et al., 2017; Hou et al., 2022). Eriksson et al. (2002) reported a reduction of enzyme adsorption to pretreated lignocellulosic biomass when Tween 20 was added during enzymatic hydrolysis. The study proposes that the positive effects come from the interaction of the exposed lignin with the hydrophilic part of the surfactant, making it inaccessible to cellulolytic enzymes (Eriksson et al., 2002; Alkasrawi et al., 2003). The original MRS medium has 1% Tween 80, which was withdrawn for medium simplification (Yi et al., 2016). However, this surfactant has been shown to increase nutrient intake by improving cell wall permeability (Nielsen et al., 2016). Tween 80 was then added to the cultivation to assess its effect on D-LA production. The addition of this surfactant had a positive effect on the SSF process by reducing the lag phase from 48 to 24 h. This might either suggest a possible interaction between the cells and the surface of the pretreated softwood solids be the source for this inhibitory effect or an improvement of the nutrient intake by the cells. Further study is required to fully understand the mechanism of action of Tween 80 in the cultivation. Non-etheless, a lag phase of 24 h is still too long for possible industrial production of D-LA. The present study focused on a way to overcome this inhibition, to shorten the lag phase in the presence of pretreated softwood solids. The specific nature of the solid-cell interaction remains to be elucidated.

*P. acidilactici* ZP26 has been reported to produce EPS in association with growth (Liu et al., 2015; Campos et al., 2021). When in the late exponential or stationary phase, the flocculation of the culture caused by EPS production is evident. It was hypothesized a culture that has started to produce EPS, which is both secreted to the medium and retained in the cellular membrane for cell protection (Korcz and Varga, 2021), might be adsorbed to the pretreated wood (Santore, 2022), and thereby showing negative effects on D-LA metabolism. The interaction between biofilms and wood might change not only because of the increase in the number of cells that do not attach to the biofilms after a certain density but also might be related to changes in the physiological state of the cells, which might lead to cell disaggregation from the biofilm and subsequent improvement of mass transfer and metabolism (Santore, 2022). Since EPS production is not observed in an early growth stage, a study was conducted where the culture was initiated in a synthetic medium supplemented with glucose until it reached an early exponential phase, when pretreated solids were added to the bioreactor, with and without enzyme addition (Figure 2). The lag phase decreased from more than 40 h to less than 2 h when the solids were added after 3 h of fermentation. These results showed that the use of pretreated softwood solids for the production of D-LA was possible without a long start-up phase, as long as these solids are only added to the reactor after cells are grown to an early exponential phase.

## 5 Conclusion

A longer lag phase was observed in SSF experiments with pretreated softwood solids compared with previous experiments using the liquid fraction of the steam-pretreated softwood. It was found that the lag phase could be reduced by adding the pretreated solids and starting the SSF when cells were in an early stage of exponential growth. The significant decrease demonstrated in this work is substantial when designing a process for an industrial scale bioprocess.

It is inferred that the decrease in inhibitory effect was likely due to a change in the interaction between the cells and the solid particles, possibly due to a changed EPS content as a function of the physiological status of the culture. It is necessary to study further the SSF of softwood for the production of D-LA by optimizing the enzymatic hydrolysis conditions to achieve maximum hydrolysis yield, using the liquid fraction instead of defined glucose to start the culture before the addition of the pretreated solids, and following downstream processing for product purification, for example. Nevertheless, this study sheds light on a type of inhibition that is not commonly addressed (cell-solid interaction) and opens the door to the utilization of softwood for biochemical production through SSF without the need for a detoxification step to decrease inhibition effects.

## Data Availability

The original contributions presented in the study are included in the article/Supplementary Material, further inquiries can be directed to the corresponding author.
