# Supplementary material for: Overcoming extended lag phase on optically pure lactic acid production from pretreated softwood solids
Source: Front Bioeng Biotechnol. 2023 Sep 7;11:1248441. doi: 10.3389/fbioe.2023.1248441 (PMC10513496; doi:10.3389/fbioe.2023.1248441)
Supplement: Supplementary file 1 [file Image1.pdf]

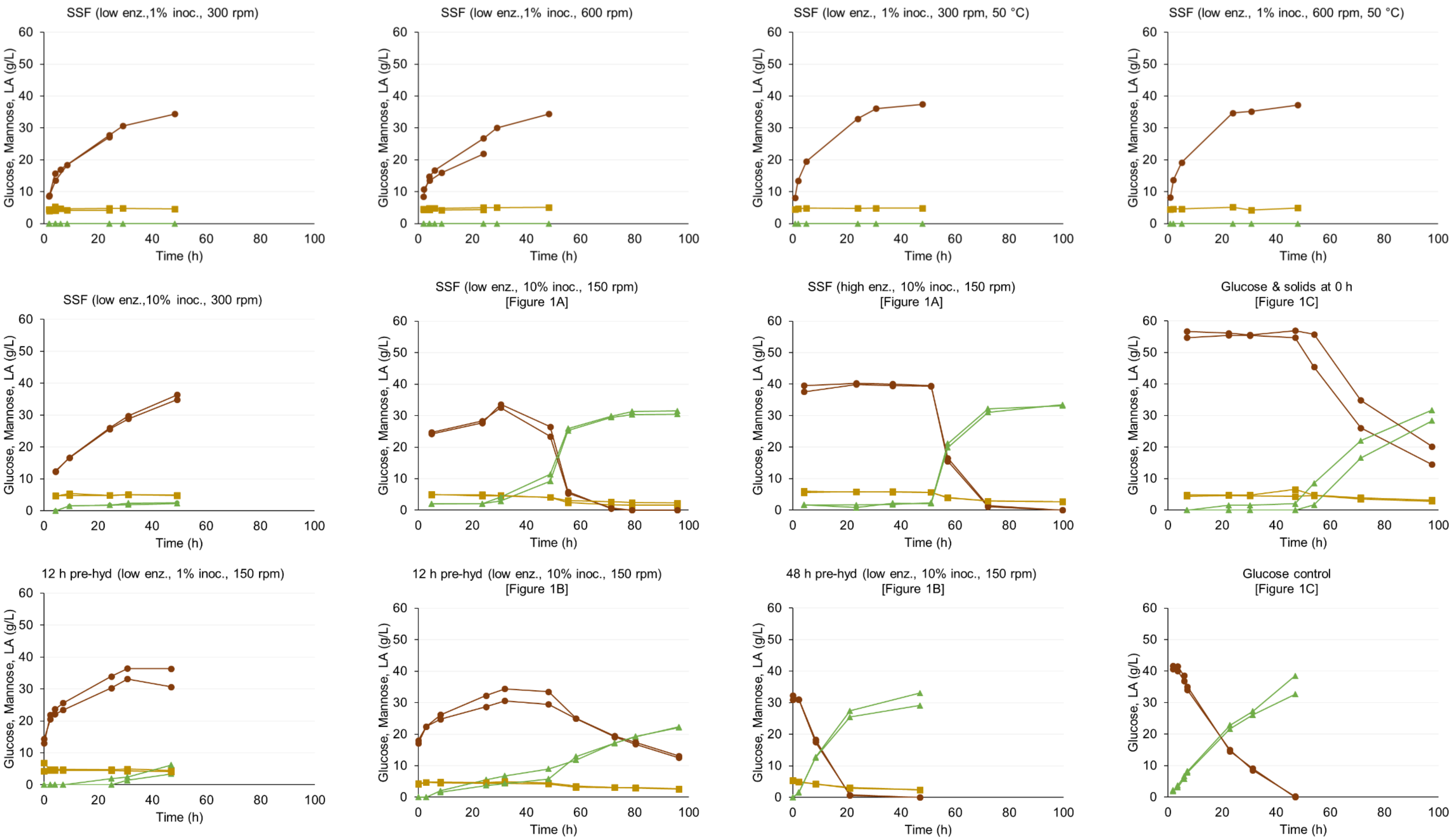

Figure 1. Raw data behind Figure 1 of the manuscript, along with other cultivations made during this study. The title of each graph and the references in [ ] identify the correspondent graph in the manuscript. Legend: glucose (maroon circles), mannose (gold squares), and LA (green triangles).

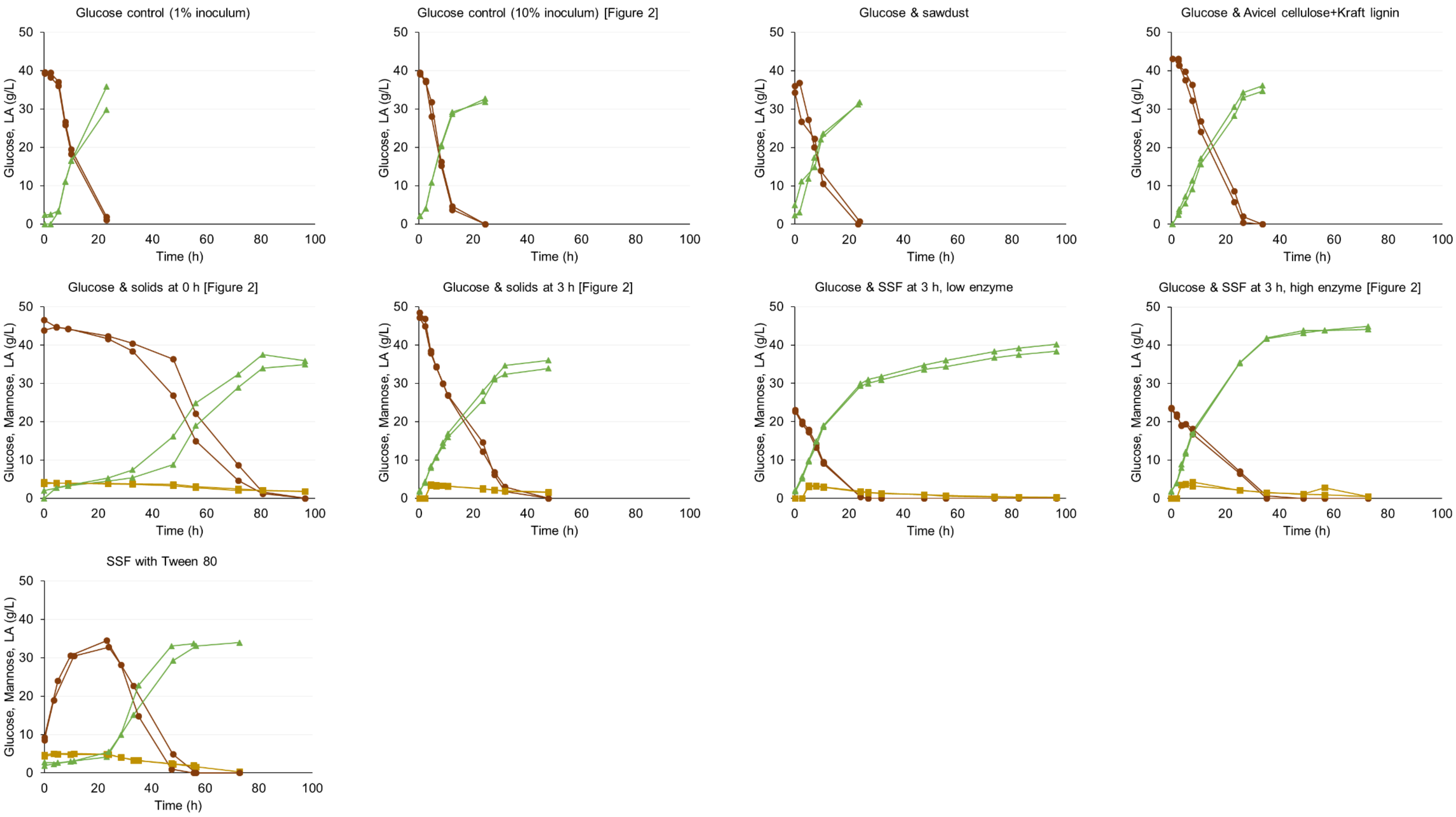

Figure 2. Raw data behind Figure 2 of the manuscript, along with other cultivations made during this study. The title of each graph and the references in [ ] identify the correspondent graph in the manuscript. Legend: glucose (maroon circles), mannose (gold squares), and LA (green triangles).
